# Supplementary material for: Towards an open analysis ecosystem for Plasmodium genomic epidemiology
Source: medRxiv. 2025 Apr 1:2025.04.01.25325032. Preprint. [Version 1] doi: 10.1101/2025.04.01.25325032 (PMC11998834; doi:10.1101/2025.04.01.25325032)
Supplement: Supplement 3 [file NIHPP2025.04.01.25325032v1-supplement-3.pdf]

medRxiv preprint doi: https://doi.org/10.1101/2025.04.01.25325032; this version posted April 1, 2025. The copyright holder for this preprint
(which was not certified by peer review) is the author/funder, who has granted medRxiv a license to display the preprint in perpetuity.
It is made available under a CC-BY 4.0 International license .

Table 2: Tool landscaping matrix *(attached as .xlsx)*

medRxiv preprint doi: https://doi.org/10.1101/2025.04.01.25325032; this version posted April 1, 2025. The copyright holder for this preprint (which was not certified by peer review) is the author/funder, who has granted medRxiv a license to display the preprint in perpetuity.
It is made available under a CC-BY 4.0 International license .

Supplementary material

Supplementary Table 1: All tools landscaping matrix *(attached as .xlsx)*

Supplementary Table 2: "Nice to have" software standards

Criteria	Type	Notes
**User-facing**		
Informative error handling	binary	
Multiple languages for tutorials	binary	
Uses standard data input formats	binary	Eg decreases the need for the user to wrangle data into required input format
**Developer-facing**		
Minimal dependencies	binary	
Computationally efficient	binary	
Modular code	binary	Eg split into functions
Well-annotated code	binary	
